# Effect of positive reinforcement nursing combined with standardized detailed care in emergency infusion patients

**DOI:** 10.1097/MD.0000000000043456

**Published:** 2025-07-25

**Authors:** Min Liu, Linfang Li, Yuqing Xia, Minfei Huang, Xueke Fan, Yihui Shen

**Affiliations:** a Emergency Department of Affiliated Hospital of Jiangnan University, Wuxi, Jiangsu Province, China; b EICU of Affiliated Hospital of Jiangnan University, Wuxi, Jiangsu Province, China; c Outpatient Department of Affiliated Hospital of Jiangnan University, Wuxi, Jiangsu Province, China.

**Keywords:** emergency, infusion, positive reinforcement nursing

## Abstract

Intravenous infusion is a common and critical treatment modality in the emergency department. Optimizing infusion nursing quality is essential for improving patient clinical outcomes. This study aims to evaluate the efficacy of positive reinforcement nursing interventions in patients receiving emergency infusions. This retrospective study included 971 patients who underwent infusion therapy in the emergency department from February 2023 to February 2024. Based on different nursing interventions, patients were divided into the experimental group (476 patients receiving positive reinforcement nursing) and the control group (495 patients receiving routine nursing). After propensity score matching, each group comprised 320 patients. Data collected included general patient information, Modified Early Warning Score, Self-Rating Anxiety Scale, Self-Rating Depression Scale, Visual Analog Scale for pain, nursing satisfaction, and Nottingham Health Profile quality of life scores. Statistical analyses were performed using SPSS software, with a significance level set at *P* < .05. Post-matching, there were no significant differences in baseline characteristics between the 2 groups (*P* > .05). On the first and third days of infusion, the experimental group exhibited significantly lower Visual Analog Scale scores compared to the control group (*P* < .05). Following nursing interventions, the experimental group showed significantly lower Self-Rating Anxiety Scale and Self-Rating Depression Scale scores than the control group (*P* < .001). Regarding infusion-related adverse events, the experimental group had a significantly lower incidence of repeated punctures and redness/swelling at the puncture site compared to the control group (*P* < .05). In terms of nursing satisfaction, the experimental group scored significantly higher in nursing skills, infusion room environment, and infusion management compared to the control group (*P* < .05). For quality of life, the experimental group had significantly lower Nottingham Health Profile scores in all dimensions except social life compared to the control group (*P* < .05). Positive reinforcement nursing interventions significantly reduce pain perception in emergency infusion patients, improve their psychological state, decrease the incidence of infusion-related adverse events, and enhance nursing satisfaction and quality of life. These findings highlight the clinical value of integrating positive reinforcement strategies into infusion nursing practices in emergency settings.

## 1. Introduction

In the emergency department, intravenous (IV) therapy is a common and critical treatment modality, widely used in various clinical scenarios such as fluid supplementation, electrolyte balance correction, medication administration, and emergency interventions. Emergency infusions not only rapidly improve patients’ physiological status but also play a vital role in the early management of diseases.^[1–3]^ However, due to the complex and variable conditions of emergency patients, multiple adverse events can occur during the IV infusion process, such as infusion site infections, drug extravasation, and puncture failures. These complications not only affect treatment outcomes but also increase patient discomfort and healthcare costs. Therefore, optimizing infusion nursing quality and reducing infusion-related complications have become urgent issues that need to be addressed in the current field of emergency nursing.^[4–6]^

Current research primarily focuses on comparing routine nursing with single intervention strategies, lacking the evaluation of systematic and comprehensive nursing intervention models. Existing studies have demonstrated that positive reinforcement nursing has significant advantages in certain clinical settings.^[7]^ However, the specific mechanisms, long-term effects, and comprehensive impact of positive reinforcement nursing on the quality of life of emergency infusion patients remain inadequately explored. Consequently, there is an urgent need for systematic research to fill the existing gaps, clarifying the actual effects and application value of positive reinforcement nursing interventions in emergency infusion care.^[8]^

Based on the aforementioned background, this study proposes the following hypothesis: Compared to routine nursing, positive reinforcement nursing interventions can significantly reduce pain perception in emergency infusion patients, improve their psychological state, decrease the incidence of infusion-related adverse events, enhance nursing satisfaction, and improve patients’ quality of life. This study aims to evaluate the impact of positive reinforcement nursing interventions on pain perception, psychological state, incidence of adverse events, nursing satisfaction, and quality of life in emergency infusion patients.

## 2. Methods

### 2.1. Study design

This study was approved by the Ethics Committee of the Affiliated Hospital of Jiangnan University (Approval No. JNUAH2023-ER-021). Given the retrospective nature of the study and the use of anonymized patient data, the requirement for individual informed consent was formally waived by the Ethics Committee.

This retrospective study included patients who underwent IV infusion in the emergency department from February 2023 to February 2024. Based on the inclusion and exclusion criteria, a total of 971 valid cases were enrolled. According to the differences in nursing interventions received during previous infusions, patients were divided into the experimental group and the control group. The experimental group consisted of 476 patients who previously received positive reinforcement nursing interventions combined with standardized detailed nursing care, while the control group comprised 495 patients who previously received routine nursing care.

Inclusion criteria: Patients aged 18 years and above receiving IV infusions in the emergency department; patients at their first emergency visit who had not yet received any pharmacological treatments; patients who completed a full course of IV therapy; patients who received comprehensive combined nursing or routine nursing during infusion; patients who were conscious and able to cooperate with nursing staff for treatment and assessment; patients with complete assessment records.

Exclusion criteria: Patients with critical conditions such as myocardial infarction or stroke who required emergency resuscitation and advanced life support; patients with advanced malignant tumors or other diseases with poor prognosis, where the specific nature of the disease precludes benefits from nursing interventions; patients with a history of mental illness or severe cognitive impairment, who were unable to follow nursing instructions or self-report discomfort; patients allergic to infusion medications or materials used during the nursing process; pregnant or lactating patients, or those unable to complete the full course of nursing interventions and assessments for other reasons.^[9–12]^

### 2.2. Nursing methods

#### 2.2.1. Routine nursing

Verify the patient’s basic information, perform venipuncture following aseptic technique protocols, and conduct frequent monitoring of the patient during the infusion.

#### 2.2.2. Combined nursing

(1) Positive reinforcement nursing interventions:
  (a) Health education: Educate patients and their families about the purpose and precautions of IV infusion. Remind them to observe any abnormal conditions during the infusion process, promptly report and address issues, ensuring that patients understand and cooperate with the infusion procedure.
  (b) Comfort care: Assist patients in adjusting to comfortable positions to ensure smooth infusion. Increase the frequency of monitoring, promptly inquire about patient needs, and observe the needle and infusion site for normalcy.
  (c) Post-infusion care: After the infusion is completed, remove the needle and apply pressure to the puncture site to prevent bruising. For patients with poor vascular conditions, apply a warm compress to promote vascular recovery.
(2) Standardized detailed nursing:
  1. Environment optimization: Nursing staff should disinfect areas such as the emergency waiting corridors, consultation rooms, and IV infusion treatment rooms every 4 hours to ensure the internal air of corridors and wards remains fresh and odor-free. Maintain the relative humidity in the infusion treatment room between 50% and 60%, and the temperature between 24°C and 26°C. Ensure the infusion area is clean, well-ventilated, and maintains appropriate temperature and humidity. Provide essential facilities, such as drinking water equipment, to ensure that patients receive treatment in a comfortable environment.
  2. Health education: Actively communicate with patients, providing detailed explanations of their conditions, the infusion process, and medication information. Remind patients not to adjust the infusion rate on their own, answer their questions, and ensure they have a thorough understanding of their treatment.
  3. Process optimization: Strictly verify patient identity, medical orders, and medications. After ensuring the safety of the infusion solution, perform aseptic operations and avoid selecting puncture sites that are prone to complications.
  4. Increased monitoring: During the infusion, regularly check the drip rate, puncture site, and patient responses. Promptly address any abnormalities to ensure patient safety.

### 2.3. Data collection

#### 2.3.1. General data collection

Collected general information included age, gender, body mass index (BMI), education level, disease type, medical history, and records of adverse infusion events.

#### 2.3.2. MEWS score

The Modified Early Warning Score (MEWS) is a vital sign-based scoring system comprising 5 components: respiratory rate, heart rate, systolic blood pressure, body temperature, and level of consciousness (Alert-Verbal-Pain-Unresponsive scale). Each component is scored based on specific numerical ranges, with higher total scores indicating more severe clinical conditions. The maximum possible score is 14 points.

#### 2.3.3. Anxiety and depression scores

SAS: The Self-Rating Anxiety Scale (SAS) assesses patients’ anxiety symptoms over the past week, consisting of 20 items. Each item is scored from 1 (no symptoms) to 4 (severe symptoms), with standardized total scores ranging from 20 to 80. Higher scores indicate greater levels of anxiety.^[13]^

SDS: The Self-Rating Depression Scale (SDS) measures patients’ depressive symptoms and also includes 20 items, each scored from 1 to 4. After standardization, total scores range from 20 to 80, with higher scores reflecting more severe depressive symptoms.^[14]^

#### 2.3.4. Nursing satisfaction score

Nursing satisfaction was assessed 3 days post-intervention using a questionnaire-based scoring method. The evaluation comprised 4 items with a total of 25 questions, yielding a maximum score of 100 points. Each of the 4 domains was allocated 25 points.

#### 2.3.5. Quality of life score

Quality of life was evaluated using the Nottingham Health Profile (NHP) both before the intervention and 3 days after. The NHP encompasses 6 dimensions: physical activity, pain, emotional reactions, social life, sleep, and energy levels. Each dimension has a maximum score of 100 points. Lower scores indicate better quality of life.

#### 2.3.6. Statistical analysis

Comprehensive statistical analyses were performed using SPSS software (IBM Corp., Armonk). For continuous variables, the Shapiro–Wilk test was first conducted to assess normality. Normally distributed data were presented as mean ± standard deviation and compared between groups using independent samples *t* tests. Categorical variables were expressed as frequencies or percentages and compared using the Chi-square test. A *P* value of <0.05 was considered statistically significant, indicating that the observed differences were unlikely due to random variation.

To reduce potential confounding and ensure comparability between groups, a propensity score matching (PSM) analysis was performed using a 1:1 nearest-neighbor matching method without replacement and with a caliper of 0.02. The propensity score model included the following baseline covariates: age, gender, BMI, education level, disease type, MEWS, infusion duration, and history of diabetes or hypertension. These variables were selected based on clinical relevance and their potential association with both the exposure (nursing intervention type) and the outcomes.

## 3. Results

### 3.1. Matching of basic information of emergency infusion patients

**Figure 1.**
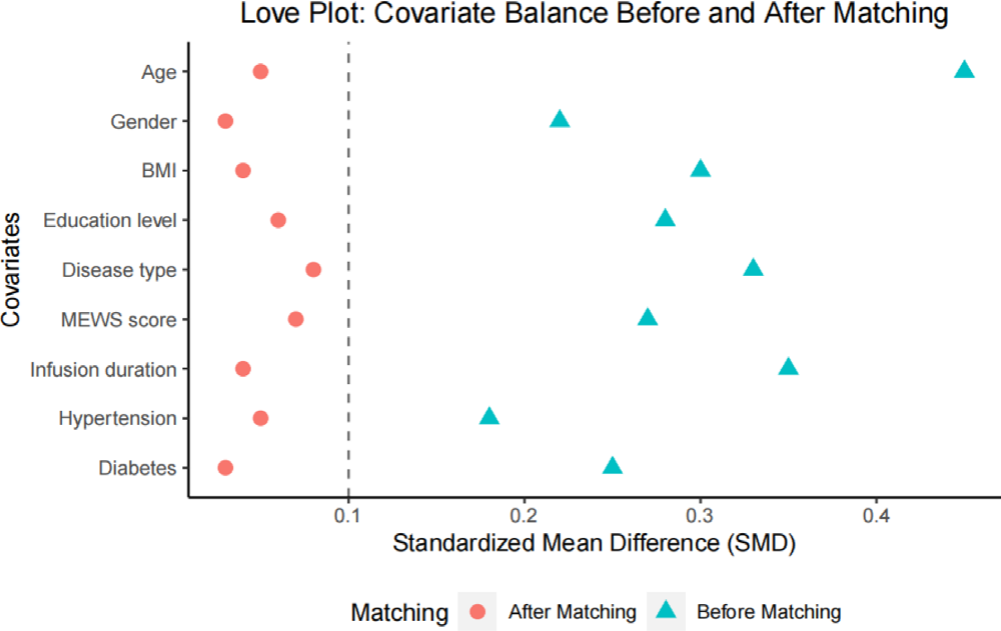
Love plot showing covariate balance before and after propensity score matching.

To ensure the scientific validity and reliability of the subsequent study results and to minimize the interference of confounding factors, we conducted statistical analysis and PSM on the basic characteristics of the 2 patient groups. As shown in Table 1, the initial analysis revealed significant differences between the 2 groups in variables such as age, BMI, education level, disease type, MEWS, infusion duration, and prevalence of diabetes. After applying the PSM method, there were no statistically significant differences in these basic characteristics between the matched groups (*P* > .05). This indicates that these variables were effectively controlled post-matching. By employing this matching method, we ensured the baseline characteristics of the 2 patient groups were balanced, thereby providing a more robust statistical foundation for further analysis of the intervention effects. Figure 1 presents the standardized mean differences for covariates before and after matching. The plot demonstrates substantial improvement in covariate balance following PSM, with all post-matching standardized mean differences falling below the conventional threshold of 0.1.

**Table 1:** Basic information.

Variables	Before matching			After matching		
	Experimental group	Control group	*P* value	Experimental group	Control group	*P* value
Total number of individuals	476	495		320	320	
Age (yr)	45.16 ± 9.31	5.18 ± 8.21	<.001	48.55 ± 7.64	48.61 ± 7.88	.774
Gender			.269			.411
Male	318	347		199	209	
Female	158	148		121	111	
BMI (kg/m^2^)	22.16 ± 4.51	2.13 ± 4.69	.031	21.57 ± 4.67	21.97 ± 5.12	.519
Educational level			.028			.114
Junior high school and below	299	344		165	145	
High school and above	177	151		155	175	
Disease type			<.001			.822
Urinary tract infection	51	82		45	48	
Cholecystitis	71	53		61	69	
Appendicitis	89	96		85	78	
Acute gastroenteritis	136	169		63	67	
Other diseases	129	95		66	58	
MEWS score	5.13 ± 1.01	4.95 ± .98	.041	5.23 ± .97	5.19 ± 1.01	.116
Infusion days	6.15 ± 3.34	7.13 ± 2.95	.003	6.91 ± 3.11	7.02 ± 2.98	.203
Previous history						
Hypertension	89	71	.067	61	65	.691
Diabetes	41	20	.003	29	19	.133
Propensity score($\bar{X}\pm S$)	0.43 ± 0.15		<.01	.06 ± .09		.110

BMI = body mass index, MEWS = Modified Early Warning Score.

### 3.2. Comparison of Visual Analog Scale (VAS) scores between groups at different time points

We focused on comparing the primary assessment indicator of infusion nursing – pain perception – using the VAS for evaluation. As shown in Table 2, prior to infusion nursing, there was no significant difference in VAS scores between the 2 groups (*P* > .05). On the first and third days of infusion nursing, there were significant.

**Table 2:** Comparison of VAS between the 2 groups.

	Pre-care	Infusion day 1	Infusion day 2	Infusion day 3
Experimental group (n = 320)	8.16 ± 2.36	6.09 ± 2.01*	4.03 ± 1.98*	3.00 ± 2.01*
Control group (n = 320)	8.21 ± 3.11	6.98 ± 1.95*	4.16 ± 0.97*	3.89 ± 1.89*
*T* value	0.131	0.714	0.023	0.702
*P* value	.218	.021	.065	.043

VAS = Visual Analog Scale.

* It indicated that there was significant difference between the 2 groups (*P* < .05).

### 3.3. Comparison of SAS and SDS scores between groups

We compared the SAS and SDS scores between the 2 groups of patients, as shown in Table 3. Prior to nursing interventions, there were no significant differences in SAS and SDS scores between the 2 groups (*P* > .05). After the nursing interventions, the experimental group had SAS and SDS scores of 22.47 and 25.99, respectively, compared to 35.24 and 35.18 in the control group. Both differences were statistically significant (*P* < .05). These results indicate that the nursing interventions in the experimental group were significantly more effective in alleviating patients’ anxiety and depression, thereby enhancing their psychological well-being.

**Table 3:** Comparison of SAS and SDS between the 2 groups.

Group	SAS		SDS	
	Pre-care	After care	Pre-care	After care
Experimental group (n = 320)	57.16 ± 6.13	22.47 ± 4.91*	56.39 ± 6.91	25.99 ± 3.32*
Control group (n = 320)	57.51 ± 6.01	35.24 ± 5.21*	56.22 ± 6.77	35.18 ± 4.14*
*T* value	0.415	12.163	0.106	9.851
*P* value	.455	<.001	.534	<.001

SAS = Self-Rating Anxiety Scale, SDS = Self-Rating Depression Scale.

* It indicated that there was significant difference between the 2 groups (*P* < .05).

### 3.4. Comparison of infusion-related adverse events between groups

Due to the acute nature of patients in the emergency department, infusion-related adverse events are most likely to occur during IV infusions. Therefore, we evaluated the incidence of such adverse events. As shown in Table 4, significant differences were observed between the 2 groups in the composition of adverse events, specifically in repeated punctures and redness/swelling at the puncture site. In the experimental group, there were 11 cases of repeated punctures compared to 29 cases in the control group (*P* < .05). Additionally, redness and swelling at the puncture site occurred in 21 cases in the experimental group and 36 cases in the control group (*P* < .05). However, there were no significant differences between the 2 groups regarding drug extravasation and accidental dislodgement (*P* > .05).

**Table 4:** Comparison of infusion adverse events between the 2 groups.

	Accidental extubation	Drug extravasation	Repeated puncture	The puncture is red and painful
Experimental group (n = 320)	2	7	11	21
Control group (n = 320)	5	6	29	36
*X*^2^ value	0.014	0.031	0.501	0.392
*P* value	.879	.701	.021	.047

### 3.5. Comparison of nursing satisfaction between groups

Additionally, we compared the nursing satisfaction between the 2 groups of patients, as shown in Table 5. Nursing satisfaction was evaluated across 4 aspects. Firstly, there was no significant difference between the 2 groups in the aspect of work attitude, with scores of 21.36 and 21.51 for the experimental and control groups, respectively (*P* > .05). However, significant differences were observed between the 2 groups in nursing skills, infusion room environment, and infusion control management (*P* < .05). Notably, in the area of infusion control management, the experimental group scored significantly higher than the control group, with scores of 23.36 and 20.19, respectively.

**Table 5:** Comparison of nursing satisfaction between the 2 groups.

	Work attitude	Nursing technique	Infusion room environment	Infusion management
Experimental group (n = 320)	21.36 ± 4.12	20.59 ± 2.98	19.26 ± 3.11	23.36 ± 2.09
Control group (n = 320)	21.51 ± 3.69	18.95 ± 2.45	17.33 ± 2.90	20.19 ± 1.98
T value	0.251	2.155	1.974	2.358
*P* value	.770	.030	.021	.011

### 3.6. Comparison of NHP scores between groups

Finally, we compared the NHP quality of life scores between the 2 groups of patients, as shown in Table 6. Quality of life was assessed across 6 dimensions: physical activity, pain, emotional reactions, social life, sleep, and energy levels. Except for the social life dimension, where no significant difference was observed between the 2 groups (*P* > .05), all other 5 dimensions showed significant differences (*P* < .05). The experimental group had significantly lower scores compared to the control group in these dimensions, indicating greater improvements in quality of life. Notably, in the physical activity dimension, the experimental group scored 51.21, whereas the control group scored 63.12, demonstrating a more pronounced improvement in physical functioning among patients in the experimental group.

**Table 6:** Comparison of NHP between the 2 groups.

Group	Somatic activity		Pain		Emotional response	
	Pre-care	After care	Pre-care	After care	Pre-care	After care
Experimental group (n = 320)	71.51 ± 6.21	51.21 ± 1.96*	70.01 ± 2.31	56.15 ± 3.21*	73.26 ± 5.41	40.36 ± 3.24*
Control group (n = 320)	72.36 ± 5.21	63.12 ± 2.51*	70.03 ± 2.12	60.36 ± 2.49*	73.51 ± 6.16	52.16 ± 2.90*
T value	1.201	12.015	0.032	4.351	0.325	12.365
*P* value	.515	<.001	.748	<.001	.101	<.001
Group	Social life		Sleep		Vigor	
	Pre-care	After care	Pre-care	After care	Pre-care	After care
Experimental group (n = 85)	68.51 ± 6.21	52.35 ± 2.63*	69.94 ± 4.13	49.65 ± 3.22*	67.12 ± 3.22	50.16 ± 2.41*
Control group (n = 85)	67.20 ± 5.99	52.12 ± 3.11*	69.80 ± 3.92	55.18 ± 2.95*	67.91 ± 3.19	55.21 ± 3.01*
T value	1.521	0.100	0.136	6.154	0.815	5.136
*P* value	.512	.354	.336	<.001	.412	<.001

NHP = Nottingham Health Profile.

* It indicated that there was significant difference between the 2 groups (*P* < .05).

## 4. Discussion

IV therapy in the emergency department is one of the primary treatment modalities administered to patients upon hospital admission. Due to its direct administration route, rapid clinical efficacy, and ease of operation, IV therapy is extensively utilized in the prevention and treatment of various clinical conditions. The emergency department is one of the busiest departments in hospitals, characterized by high patient flow, a wide range of medical conditions, and substantial nursing workloads. The quality of nursing services in this department is a crucial indicator of the hospital’s reputation, garnering significant attention from hospital leadership and the public. However, as an invasive clinical nursing procedure, IV administration carries the risk of adverse events if not performed meticulously. Additionally, patients and their families may experience heightened emotions or dissatisfaction due to disease-related distress or delays in nursing care, potentially leading to nursing disputes and, in severe cases, irreversible harm to patients. Therefore, enhancing comprehensive and detailed nursing care during the pre-, intra-, and post-infusion periods for emergency patients is of paramount importance.^[13,14]^

Positive reinforcement nursing combined with standardized detailed nursing addresses the shortcomings of routine nursing by centering care around the patient’s specific needs. This approach strictly adheres to aseptic principles of IV infusion and formulates specific, high-quality nursing interventions to ensure effective IV therapy.

This study conducted a retrospective analysis to evaluate the clinical efficacy of positive reinforcement nursing combined with standardized detailed nursing in emergency infusion patients. The results demonstrated that the experimental group significantly outperformed the control group across multiple indicators.^[15,16]^ Specifically, patients in the experimental group exhibited lower pain perception (VAS scores), reduced anxiety (SAS scores), decreased depression (SDS scores), lower incidence of infusion-related adverse events, higher nursing satisfaction, and improved quality of life (NHP scores) compared to those receiving routine nursing care. These findings support the effectiveness of positive reinforcement nursing interventions in enhancing clinical outcomes for emergency infusion patients.^[17,18]^

Positive reinforcement nursing combined with standardized detailed nursing significantly alleviated patients’ pain perception. On the first and third days of infusion care, the experimental group had notably lower VAS scores than the control group, indicating effective pain relief. Health education and comfort care likely enhanced patients’ understanding and cooperation with the infusion process, reducing pain perception triggered by uncertainty and anxiety. Furthermore, the experimental group showed significantly lower SAS and SDS scores than the control group, suggesting that the combined nursing interventions effectively mitigated anxiety and depression, thereby improving patients’ psychological well-being.

The reduction in infusion-related complications in the experimental group aligns with existing literature, which indicates that standardized nursing practices can decrease the incidence of such adverse events.^[19]^ By optimizing puncture techniques and strengthening care at the infusion site, positive reinforcement nursing effectively minimized complications like repeated punctures and redness/swelling at the puncture site. These improvements contribute to enhanced patient safety and comfort during IV therapy.

In terms of quality of life, the experimental group demonstrated significant improvements in 5 out of 6 NHP dimensions compared to the control group, except for social life, where no significant difference was observed. Notably, the physical activity dimension showed a substantial decrease in scores for the experimental group, reflecting better physical functioning. This improvement is likely due to effective pain management and comfort care, which facilitate the recovery of patients’ physical functions.

This study confirms the multifaceted advantages of positive reinforcement nursing interventions in managing emergency infusion patients. It underscores the importance of systematic and personalized nursing measures. By implementing comprehensive nursing strategies such as health education, comfort care, and environmental optimization, not only can physiological and psychological distress be alleviated, but the overall quality of nursing services and patient satisfaction can also be enhanced. These findings have significant implications for improving the efficiency and effectiveness of nursing work in emergency departments.^[20]^

## 5. Limitations

As a retrospective study, this research has inherent limitations, including potential selection bias and information bias, which make it difficult to completely eliminate the influence of confounding factors. To improve internal validity, the study was conducted at a single medical institution. However, this single-center design may limit the external validity and generalizability of the findings to other healthcare settings, especially those with different patient demographics, staffing patterns, or nursing protocols. Furthermore, differences in nursing execution across staff members may have introduced variability in intervention fidelity. To address these limitations and enhance both internal and external validity, future research should consider conducting multi-center randomized controlled trials involving diverse populations and standardized intervention protocols across institutions. Such studies would provide more robust evidence for the broader applicability and scalability of positive reinforcement nursing interventions in emergency care.

## 6. Conclusion

This study demonstrates that positive reinforcement nursing interventions have significant clinical advantages in emergency infusion patients, including reducing pain perception, improving psychological state, decreasing infusion-related adverse events, enhancing nursing satisfaction, and improving quality of life. It emphasizes the importance of comprehensive nursing measures in enhancing patient care outcomes. These interventions can be considered for clinical promotion, and continuous optimization and improvement of nursing care for emergency patients should be pursued.

## Author contributions

**Conceptualization:** Min Liu, Linfang Li, Yuqing Xia, Minfei Huang, Yihui Shen.

**Data curation:** Min Liu, Linfang Li, Xueke Fan, Yihui Shen.

**Formal analysis:** Min Liu.

**Funding acquisition:** Min Liu.

**Investigation:** Linfang Li, Yuqing Xia, Xueke Fan, Yihui Shen.

**Methodology:** Min Liu, Linfang Li, Yuqing Xia, Xueke Fan, Yihui Shen.

**Supervision:** Minfei Huang.

**Validation:** Linfang Li, Minfei Huang.

**Visualization:** Linfang Li, Minfei Huang.

**Writing – original draft:** Min Liu, Yihui Shen.

**Writing – review & editing:** Min Liu, Yihui Shen.
